# Prevalence and seasonal dynamics of gastrointestinal nematodes of domestic fowls (*Gallus gallus domesticus*) in Kashmir, India

**DOI:** 10.5455/javar.2021.h533

**Published:** 2021-09-20

**Authors:** Ishrat Ara, Humira Khan, Tanveer Syed, Bilal Bhat

**Affiliations:** 1 Department of Zoology, University of Kashmir, Srinagar, India; 2 Sher-e-Kashmir University of Agricultural Sciences & Technology of Kashmir, Jammu and Kashmir, India

**Keywords:** Domestic fowl, Kashmir, nematodes, parasitic load, prevalence

## Abstract

**Objective:**

The current study was undertaken to evaluate the seasonal dependency and prevalence of gastrointestinal roundworms (nematodes) infecting domestic fowls (*Gallus gallus domesticus*) in Kashmir.

**Materials and Methods:**

From August 2017 through July 2019, the investigation was undertaken during each of the four seasons. We tested 400 guts obtained from varied places around the Kashmir valley for nematode infestation. The nematodes found within the digestive tract were collected and identified using a variety of identification keys under the microscope. Statistical Package for the Social Sciences was used to analyze the data (version 20). Chi-square (*χ*^2^) test was carried out to analyze the sample data.

**Results:**

196 hosts were infected with various nematodes, indicating an overall prevalence of 49% (196/400). The findings revealed that the prevalence of *Ascaridia galli* was 32.97% (61/185) in the first year and 35.34% (76/215) in the second year. *Heterakis gallinarum* had a prevalence rate of 20.80% (38/185) in the first year and 24.18% (52/215) in the second year, whereas *Capillaria* spp. had a prevalence rate of 10.81% (20/185) in the first year and 12.55% (27/215) in the second year. The overall prevalence of *A. galli* was determined to be 34.25% in both years (August 2017–July 2019), with a mean intensity of 4.86. Summer months had the highest parasitic load. *Heterakis gallinarum* had a prevalence rate of 22.5% and a mean intensity of 26.83. Summer was shown to have the most considerable parasitic burden. *Capillaria* spp. had an overall prevalence of 11.75% and a mean intensity of 4.59; autumn had the highest parasite load. The most abundant species was identified as *A. galli*. It was shown that there is a significant (*p* < 0.01) link between seasonality and helminth parasite prevalence.

**Conclusion:**

The study’s findings indicate that these gastrointestinal nematodes are ubiquitous throughout the year, but are particularly abundant in the summer and fall seasons among domestic poultry in the study area. This study on the prevalence of gastrointestinal nematodes in *Gallus gallus domesticus* demonstrates the seasonality of infection rates and also offers various methods and techniques for framing effective strategies for controlling these helminthes to maximize profit from backyard chicken farming.

## Introduction

Poultry has been recognized for thousands of years to provide meat and eggs, which are considered the two primary sources of animal protein for humans. India has a large poultry population of 498 million birds, which is growing at an average annual pace of 8%–10%. India is the third largest producer of eggs and the sixth largest producer of broiler meat [1]. Poultry production is constrained by a number of constraints, the most significant of which are illnesses, including bacterial, viral, and parasite infections [2]. Domestic chickens often consume a variety of foods, including grains (cereals), fruits, and insects that may contain the eggs or larval stages of certain helminth parasites, predisposing them to various parasitic illnesses, most notably gastrointestinal parasites [3]. 

Gastrointestinal parasites are an important factor in the decline of domestic fowl welfare [4]. Helminth parasites are generally seen in unfenced poultry around the world. The reason for the frequent recurrence of roundworm infections in an unfenced poultry system is mostly due to close contact with their feces, which ensures the completion of the nematodes’ direct life cycle via the effective fecal–oral transmission route [5]. As a result, numerous studies conducted in various parts of the world have revealed a high prevalence of chicken contamination with gastrointestinal helminths; in this context, helminths are regarded as a significant cause of bad health and decreased poultry yield [6]. 

Roundworms are a significant group among helminth parasites of poultry birds, in terms of both species and the amount of damage they inflict. *Ascaridia*, *Heterakis*, and *Capillaria* are the three major genera of roundworms that infect domestic chickens [7]. Throughout the year, many types of gastrointestinal parasites are prevalent in backyard poultry [8]. Although researchers in the Kashmir valley, such as Dar and Tanvir et al. [9], Tanveer et al. [10], and Salam [11], have conducted extensive work on helminth parasites of birds, there is still a knowledge gap about certain roundworms that infect domestic fowls in the region. *Capillaria* sp. is one such species that is listed in our study. We present 2-year prevalence and mean intensity data with seasonal change for roundworms, including *Capillaria* spp. that has not been documented previously in this location. Thus, the purpose of this study was to determine the prevalence rate and seasonal distribution of nematodes infecting domestic fowls in the Kashmir region, which would aid in developing subsequent control measures and preventing economic losses to our indigenous chicken business.

## Materials and Methods

### Study area and methods

From August 2017 to July 2019, the study was conducted in Jammu and Kashmir’s Kashmir province. At an elevation of 1,583 meters above sea level in the Himalayas between 34°20ʹ–34°36ʹ N latitudes and 74°82ʹ–74°85ʹ E longitudes [12], Kashmir Valley’s climate is moderate; it is usually cool in the spring and fall, slightly hot in the summer, and cold in the winter. For a period of 2 years, a total of 400 guts from local backyard chickens were collected from various marketplaces throughout the Kashmir valley. Our survey sample size was determined using the following formula [13]:

$$n=\overset{Z_{\alpha }P(1–P)}{\underset{d^{2}}{\;}}$$

We take here *P* = 0.5, *Z*_*α*_ = 1.96, and *d* = 0.05. This gives the sample size for our study as *n* ~ 384 and we chose *n* = 400.

### Parasite processing and identification

The gut samples were transported to the Parasitology Research Laboratory at the University of Kashmir’s Department of Zoology. Routine examinations of the collected samples for the presence of gastrointestinal parasites were carried out in accordance with the approach outlined by Fowler [14]. The recovered nematodes were initially stored in normal saline, completely cleaned, and then fixed in hot 70% ethanol. The obtained nematodes were kept in glycerin alcohol following fixation. Lactophenol was utilized to rapidly clear nematodes and Kaiser’s glycerin jelly was used to mount the worms. The prepared slides were examined closely under a light microscope at a magnification of 100× and identified using a variety of keys and books [10,15].

### Definitions

In this study, the prevalence was estimated by Thrusfield’s [16] equation: 

*P* = 100 × number of infected chickens/total number of observed chickens

The abundance is calculated as follows: 

*A* = number of parasite species isolated/total number of observed chickens

Mean intensity = total number of parasites/total number of hosts infected.

### Data analysis

The data were tabulated and analyzed using basic statistical techniques such as percentages, graphs, and chi-square test. *p* < 0.05 was considered significant at the 5% level of significance; *p* < 0.01 was considered significant at the 1% level of significance; and *p* > 0.05 was judged as statistically non-significant.

## Results

**Figure 1.**
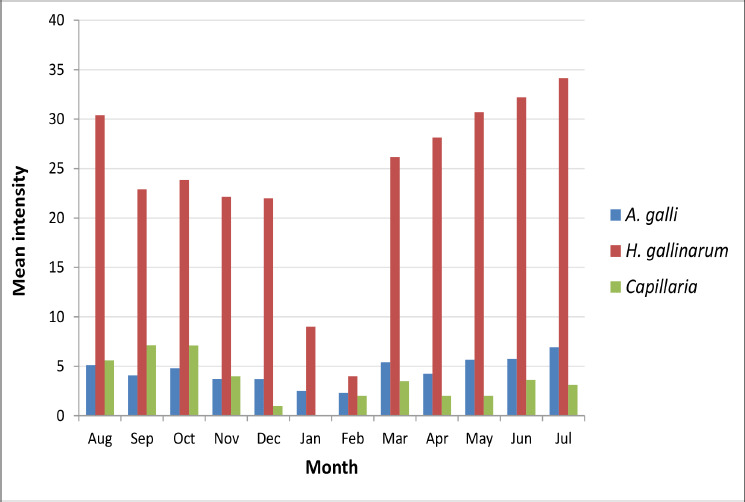
Overall mean intensity of *A. galli*, *H. gallinarum*, and *Capillaria* spp.

**Figure 2.**
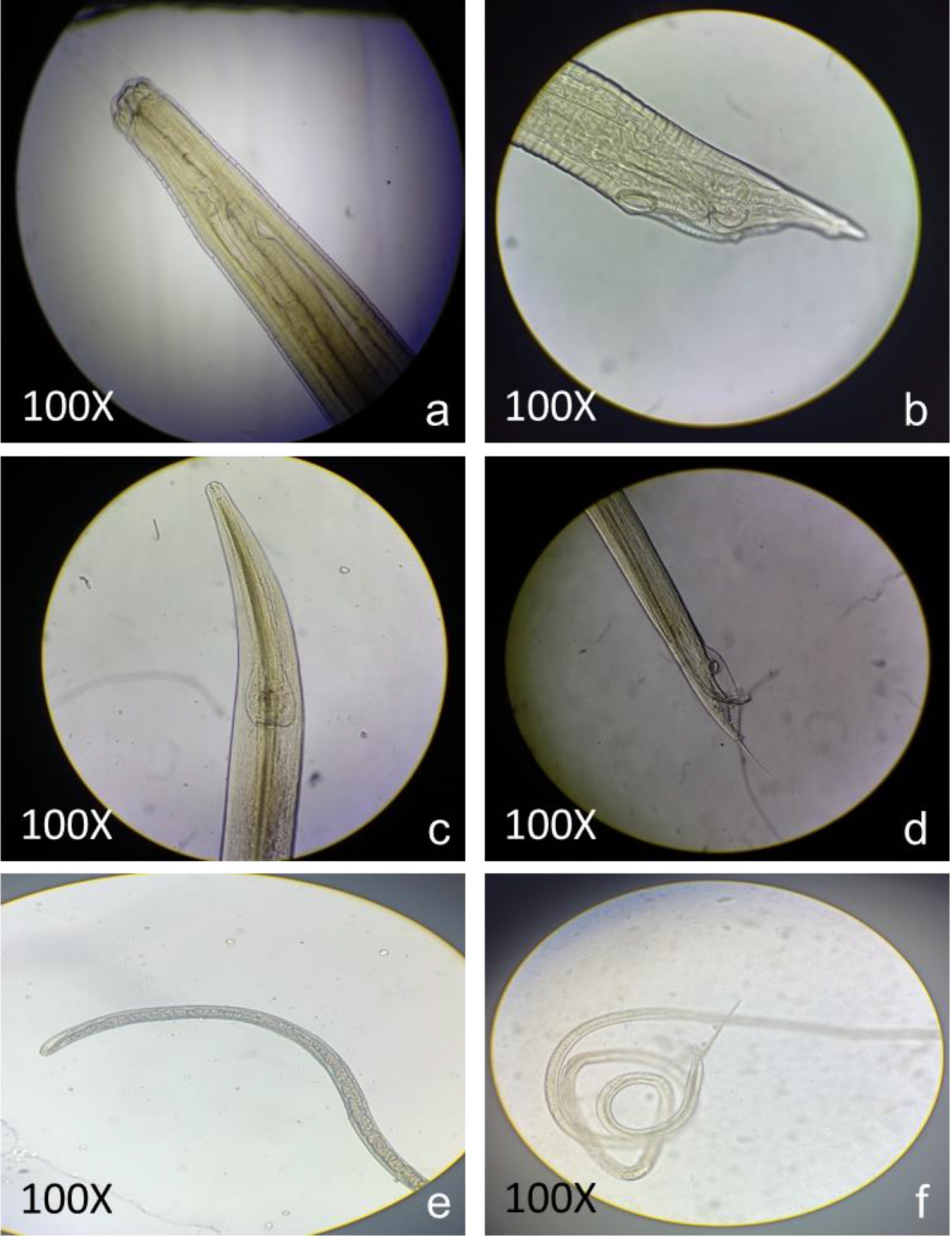
Anterior and posterior ends of collected nematodes. a) Anterior end of *A.galli*, b) posterior end of *A.galli*, c) anterior end of *H. gallinarum*, d) posterior end of *H.gallinarum*, e) anterior end of *Capillaria*, and f) posterior end of *Capillaria*.

A total of 400 gastrointestinal tracts were analyzed for nematodes during the investigation. Three nematode species, *Ascaridia galli*, *Heterakis gallinarum*, and *Capillaria* spp., were isolated from the diseased guts. For *A. galli*, the overall prevalence rate was 34.25% with a mean intensity of 4.86; for *H. gallinarum*, the prevalence rate was 22.5% with a mean intensity of 26.83; and for *Capillaria* spp., the prevalence rate was 11.75% with a mean intensity of 4.59. *Ascaridia galli* was isolated from the duodenum and *H. gallinarum* from the diseased gut’s caecum. *Capillaria* spp. was isolated from the host’s small intestine and caecum. Summer was the peak season for parasitic load in *A. galli* and *H. gallinarum*. Autumn was the season with the largest worm burden for *Capillaria* spp. Figure 1 shows the month-by-month mean intensity, while Tables 1 and 2 detail the quantitative structure of *A. galli*, *H. gallinarum*, and *Capillaria* spp. The prevalence of the collected roundworm species by season is shown in Table 3. The front and posterior ends of collected nematodes are shown in Figure 2 to differentiate these round worms.

## Discussion

During the study period, the overall prevalence of infection was found to be 49%, signifying that roundworm infection is a common problem in the region and is more or less similar to the prevalence of 45.66%, as reported by Jaiswal et al. [17]. In comparison to the current study, Sreedevi et al. [18] reported a higher frequency (63.21%) in India, and El-Dakhly et al. [19] reported a prevalence of 55.79% in Egypt. Jegede et al. [20] found a significantly lower rate (42.5%) for backyard hens in Nigeria, whereas Baboolal et al. [21] reported a rate of 10.5% for broiler chickens in Trinidad. The high frequency found in domestic fowls may be related to the type of production system, their constant contact with intermediate hosts, free-ranging management, study techniques, and parasite control strategies used in the studied areas and under the studied climatic conditions [22,23].

The birds produced from backyard poultry systems receive little or no supplemental feeds and receive no veterinary treatment; these hens are constantly scavenging and exposed to various infectious helminth stages and its intermediate hosts [24]. *Ascaridia galli* was the most abundant nematode species encountered in the study (34.25%), followed by *H. gallinarum* (22.5%) and *Capillaria* spp. (11.75%). Das et al. [8] identified *A. galli* as the most frequent nematode parasite in Meghalaya, India. Nevertheless, numerous reports indicate that *H. gallinarum* is the most frequent nematode [25]. Both Salam [11] and Eshetu et al. [26] reported a nearly same prevalence (35.35% and 35.6%) of *A. galli* in domestic fowls in Kashmir.

Sarba et al. [27] found a significant incidence of *A. galli* (69.8%) and a low prevalence of *H. gallinarum* (13.5%) in Ethiopian backyard chickens. *Heterakis gallinarum* is the most prevalent nematode in infected poultry intestinal caeca. This may be attributed to their fully developed digestive system, which provides them with a better opportunity to build a positive host–parasite interaction. *Heterakis gallinarum* infection will expose chickens to the protozoan *Histomonas meleagridis* [19]. In comparison to our investigation, Katoch et al. [28] reported a nearly identical prevalence rate (24.0%) of *H. gallinarum* in Jammu, India. We found a prevalence rate of 11.7% for *Capillaria* spp., which was greater than the percentage reported by Katoch et al. [28] in Jammu, India. There was a significant (*p* < 0.001) correlation between seasonality and prevalence of gastrointestinal nematodes. Summer and fall had the highest prevalence rates, while winters had the lowest. Fotedar and Khateeb [29] likewise reported a high prevalence of helminth infection in September and a low prevalence in December and January, noting that the prevalence and mean worm load decreased when temperature and rainfall decreased. Das et al. [8] found that infection levels were highest in summer and lowest in winter in Meghalaya. High mean temperature and relative humidity may explain the pattern of infection seen during hot and rainy months, as these conditions are favorable for the development and survival of larval/immature stages of various parasites and insects, the latter of which act as vectors/carriers for helminths, resulting in an increased availability of infective stages for the host [30]. Winters in the valley are typically snow-covered, and domestic fowls are fed indoors; also, the low winter temperature slows down the growth of parasites and their larval stages both inside the host and in the environment [31].

**Table 1.** Quantitative structure of *A. galli*, *H. gallinarum*, and *Capillaria* infectivity in domestic fowls for the year 2017–2018.

Helminth	No. of hosts examined	No. of hosts infected	No. of individuals recovered	Prevalence rate	Mean intensity	Abundance	Index of infection
*A. galli*	185	61	284	32.97%	4.65	1.53	0.50
*H. gallinarum*	185	38	1,032	20.54%	27.15	5.57	1.14
*Capillaria* spp.	185	20	106	10.81%	5.3	0.57	0.06

**Table 2.** Quantitative structure of *A. galli*, *H. gallinarum*, and *Capillaria* infectivity in domestic fowls for the year 2018–2019.

Helminth	No. of hosts examined	No. of hosts infected	No. of individuals recovered	Prevalence rate	Mean intensity	Abundance	Index of infection
*A. galli*	215	76	382	35.34%	5.02	1.77	0.62
*H. gallinarum*	215	52	1,383	24.18%	26.59	6.43	1.55
*Capillaria* spp	215	27	110	12.55%	4.07	0.51	0.06

**Table 3.** Season-wise prevalence of gastrointestinal nematodes in *Gallus gallus domesticus.*

Season	Total no. of hosts	No. of infected hosts	Prv %	No. infected hosts with particular parasitic spp. (% prevalence)					
				*A. galli*		*H. gallinarum*		*Capillaria*	
Spring	103	44	42.7	34	33.00	20	19.41	10	9.70
Summer	103	66	64.07	47	45.63	31	30.09	17	16.50
Autumn	99	66	64.07	40	40.40	30	30.30	18	18.18
Winter	95	20	21.05	16	16.84	9	9.47	2	2.10
Total	400	196	49	137	34.25	90	22.5	47	11.75
*χ* ^2^ *p*	29.469<0.01		15.438 <0.01		14.089 <0.01		14.021 <0.01	

Magwisha et al. [23] observed that nematode infection prevalence and intensity disparities might be attributable to climatic variables (temperature and humidity) in that area. According to Shukla and Mishra [32], *A. galli* is the most prevalent parasite in both domestic and exotic chicken species. Jordan and Pattison [33], Luka and Ndams [34], and Sonune [35] identified *A. galli* as the most prevalent and important chicken helminth. The observations of Hassouni and Belghyti [36] in Morocco, Permin et al. [37] in Denmark, Ashenafi and Eshetu [38] in Ethiopia, Nithiuthai et al. [39] in Bangkok, Phiri et al. [40] in central Zambia, Mwale and Masika [41] in South Africa, and Asumang et al. [42] in Ghana coincide with our study Khan et al. [25] also reported a high incidence of *H. gallinarum* in Pakistan, Ybañez et al. [43] in the Philippines, Singh and Nama [4] in Jodhpur, and Worku and Bedanie [44] in Ethiopia. Because the results are consistent with those of numerous others, the discrepancies may be attributed to the area’s environmental factors and host feeding behavior. Temperature and humidity levels affect larval development/maturation and facilitate the transmission and ingestion of infested droppings.

## Conclusion

The study demonstrates unequivocally that helminth infection is prevalent in domestic fowls and confirms the significant frequency of the worms *A. galli* and *H. gallinarum* in the Kashmir region. Additionally, the study revealed an increase in the prevalence of *Capillaria* spp. As a result of this study, future researchers will be able to design control strategies for these roundworms based on their dispersion patterns. Increased attention should be paid to poultry management and maintenance of domestic chickens that are often free-ranging. In conclusion, additional studies highlighting and controlling various elements of parasitism in poultry and increasing domestic fowl production in the region should be conducted.

## List of Abbreviations

*A. galli*, *Ascaridia galli*; *H. gallinarum*, *Heterakis gallinarum*, spp., species; *p*-value, probability value.
